# Current Uses of Bromelain in Children: A Narrative Review

**DOI:** 10.3390/children11030377

**Published:** 2024-03-21

**Authors:** Cristian Locci, Elena Chicconi, Roberto Antonucci

**Affiliations:** Pediatric Clinic, Department of Medicine, Surgery and Pharmacy, University of Sassari, 07100 Sassari, Italy; clocci@uniss.it (C.L.); elena.chicconi@aouss.it (E.C.)

**Keywords:** bromelain, *Ananas comosus*, pineapple, therapeutic effects, biomarkers, drug response, nutraceuticals, children

## Abstract

Bromelain is a complex natural mixture of sulfhydryl-containing proteolytic enzymes that can be extracted from the stem or fruit of the pineapple. This compound is considered a safe nutraceutical, has been used to treat various health problems, and is also popular as a health-promoting dietary supplement. There is continued interest in bromelain due to its remarkable therapeutic properties. The mechanism of action of bromelain appears to extend beyond its proteolytic activity as a digestive enzyme, encompassing a range of effects (mucolytic, anti-inflammatory, anticoagulant, and antiedematous effects). Little is known about the clinical use of bromelain in pediatrics, as most of the available data come from *in vitro* and animal studies, as well as a few RCTs in adults. This narrative review was aimed at highlighting the main aspects of the use of bromelain in children, which still appears to be limited compared to its potential. Relevant articles were identified through searches in MEDLINE, PubMed, and EMBASE. There is no conclusive evidence to support the use of bromelain in children, but the limited literature data suggest that its addition to standard therapy may be beneficial in treating conditions such as upper respiratory tract infections, specific dental conditions, and burns. Further studies, including RCTs in pediatric settings, are needed to better elucidate the mechanism of action and properties of bromelain in various therapeutic areas.

## 1. Introduction

Bromelain is a complex natural mixture of sulfhydryl-containing proteolytic enzymes that can be extracted from the stem or fruit of the pineapple (*Ananas comosus*), a herbaceous plant cultivated mainly in the tropical regions of the world [1]. Pineapple is the plant from which proteases are most commonly extracted [2]. Bromelain was first isolated from pineapple juice in the late 19th century and has been used in herbal medicine for many years. Bromelain has been the subject of continued interest due to its notable therapeutic properties, and research on it has been conducted worldwide for decades [3]. Its many uses are not only limited to the medicinal field but also extend to the industrial one (textile, cosmetics, food). Moreover, its production by centrifugation, ultra-filtration, and lyophilization is highly sustainable, as it is based on fruit processing waste. Recently, there has been an increase in the commercial production of bromelain extracts, contributing to the development of faster and more efficient techniques for enzyme production and purification, including recombinant DNA techniques [2,3,4].

From a pharmacological point of view, besides its proteolytic and digestive action, bromelain has been reported to have anti-inflammatory, anticoagulant, antiedematous, and antimetastatic properties. In addition, bromelain has also been found to increase antibiotic activity [5].

Bromelain is generally regarded as safe, showing beneficial therapeutic effects, low toxicity, and few side effects. However, most of the published data are based on *in vitro* and animal studies and a number of randomized controlled clinical trials in adults. Moreover, a major limitation for the clinical use of bromelain is the variable extract composition, which results in heterogeneity of findings and makes it difficult to provide evidence-based prescriptions [6]. As a result, very little is known about the effectiveness of bromelain in children. 

In many health trends, nutritional supplements containing bromelain are currently used [4], and it is included in the Italian Ministerial list of substances that can be used in dietary supplements under “other ingredients or other substances with a nutritional or physiological effect”. The same Ministry of Health recognizes the benefits of pineapple (*Ananas comosus*, *fructus*, *stipites*) in terms of drainage of body fluids, microcirculation functionality, contrast of cellulite blemishes, and digestive function [7]. To the best of our knowledge, there are no review articles in the literature focused on the pediatric use of bromelain. This narrative review was aimed at comprehensively exploring the most important aspects of current use of bromelain in children and adolescents, which still seems limited compared to its potential. 

## 2. Methods

Literature Search Strategy

A narrative review approach was chosen to collect the most relevant evidence on the use of bromelain in children. To create a comprehensive list of relevant articles and publications for inclusion, this review focused on searching the database/search engines MEDLINE, PubMed, and EMBASE. Only articles written in English and published between 1961 and 2023 were included. The following keywords were used: “bromelain”, “*Ananas comosus*”, “pineapple”, “therapeutic effects”, “nutraceuticals”, and “children”. The retrieved articles were evaluated on the basis of titles and abstracts, and those relevant to the study objective were analyzed in full text and included in this review. *In vivo* human and animal studies, adult and pediatric studies, *in vitro* studies, and relevant reviews were included. There were no restrictions regarding the type of article.

## 3. Effects of Bromelain

Bromelain is helpful in the treatment of inflammatory, post-traumatic, or post-surgical edema. These properties may be a result of its depolymerizing action on fibrin. In fact, by interacting with lipoxygenase, bromelain increases plasmin formation and therefore fibrinolytic activity; the resulting degradation of fibrin reduces edema. The antithrombotic effect depends on the reduction in thrombus formation in blood vessels. The result is improved circulation and skin tropism, a more rapid reduction in edema and pain, and an acceleration in the physiological repair process. The evidence for efficacy is relatively more consistent when considering its oral use to reduce edema and pain after dental surgery [8,9,10,11,12], while it is weaker for improving bruising and edema after dermatologic surgery [13]. A possible role in the integrative management of breast engorgement is also reported [14].

Some studies suggest that bromelain may improve blood flow through an antiplatelet effect. However, there is no solid clinical evidence to support a significant effect on the prevention and treatment of specific cardiovascular diseases [15].

Bromelain has anti-inflammatory properties by reducing the synthesis of pro-inflammatory molecules such as prostaglandin E2, prostacyclin, and thromboxanes. *In vitro* studies suggest that bromelain’s anti-inflammatory activity is due to the inhibition of bradykinin formation at the site of inflammation, which is achieved by reducing plasma kallikrein levels. Bromelain exclusively inhibits the expression of cyclooxygenase-2 (COX-2), making it an effective anti-inflammatory alternative option to non-steroidal anti-inflammatory drugs (NSAIDs), which also inhibit the COX-1 enzyme with associated side effects [1,16,17].

Bromelain reduces the migration of leukocytes and the expression of adhesion molecules (CD128) on blood vessels [16]. It also reduces the secretion of TNF-α, IL-1β, and IL-6 when immune cells are stimulated to overproduce inflammation-induced cytokines [18,19] and inhibits the activation of CD4+ lymphocytes, which maintains the inflammatory process. Furthermore, bromelain promotes a modification in the circadian rhythm of INF-γ, IL-5 and IL-10, suggesting an immunomodulatory action on T lymphocytes [20,21].

Several studies have supported the use of bromelain, either alone or in combination with other natural remedies, to relieve pain and inflammation caused by conditions such as osteoarthritis and rheumatic diseases. It may also be helpful in the treatment of autoimmune and inflammatory disorders, including multiple sclerosis, ulcerative colitis, and respiratory diseases [20,22]. In addition, bromelain has been reported to help speed recovery from musculotendinous injuries [23].

Bromelain can reduce the thickness of mucus and make it easier to cough up due to its mucolytic properties. Its proteolytic enzymes help break down peptide bonds by adding water molecules, which helps to make thick mucus more fluid. In addition, bromelain has anti-inflammatory properties that may help reduce mucus production [1,16], and exerts a significant topical effect on the respiratory mucosa [16,20].

At the gastrointestinal level, bromelain may aid digestion in individuals affected by digestive disorders or pancreatic insufficiency, especially with high-protein meals [24]. Bromelain’s ability to fight intestinal pathogens and work with antibiotics makes it helpful in treating various infectious diseases, especially those caused by bacteria, fungi, and parasites [25,26,27]. It is beneficial in the treatment of diarrhea due to its ability to reduce mucosal secretions. It also acts as an anti-adhesion agent: it prevents bacteria from attaching to intestinal cells by inhibiting the cAMP and cGMP pathways triggered by bacterial toxins. Moreover, it modifies *E. coli* receptor attachment sites in the intestinal mucosa [28,29,30,31]. 

Bromelain has been shown to increase the absorption of certain antibiotics in humans, resulting in increased blood and urine levels [32,33,34]. The combined therapy of bromelain and antibiotics has been found to be more effective than the use of antibiotics alone in treating various diseases, such as rhinosinusitis, bronchitis, pneumonia, cutaneous staphylococcal infections, cellulitis, thrombophlebitis, perirectal and rectal abscesses [35], sinusitis [36], and urinary tract infections [37]. Bromelain, rutin, and trypsin have been used as adjuvant agents in combination with antibiotics in pediatric patients with sepsis [34].

Finally, clinical evidence suggests the efficacy of bromelain as an adjuvant in the debridement of skin burns when used as a gel for topical application [38].

## 4. Pharmacological Profile

Bromelain is extracted from the pineapple stem. Sequencing of the plant cysteine endoproteinases bromelain and papain revealed that both are members of the papain family [39]. Although the pineapple plant contains other proteolytic enzymes, including ananain and comosain, bromelain remains the primary and most extensively studied of these proteolytic enzymes [40,41]. High-performance cation-exchange liquid chromatography has characterized nine proteolytic components in raw stem bromelain [19,42]. The potential therapeutic importance of bromelain results from its biochemical and pharmacological properties, and the major component of crude bromelain is a glycosylated, monomeric single protein (proteolytic enzyme), which is present together with insoluble materials, including minerals, color pigments, protease inhibitors, organic solvents, and organic acids [43,44]. Proteinases are considered to be the most active fraction, accounting for ~2% of the total proteins [19]. Bromelain is active over a pH range from 4.5 to 9.5 [5]. It can be isolated and purified using various methods. 

It has been shown that most of the physiological effects of bromelain may not result from a single proteolytic fraction, but it is likely that its beneficial effects are due to multiple factors [5]. 

The pharmacokinetic profile of bromelain remains to be elucidated due to the difficulty in determining this component in serum and the poor knowledge of its bioaccessibility and bioavailability. The efficacy of bromelain is higher when taken orally: in fact, low doses of bromelain are rapidly degraded by protease inhibitors in serum, while oral intake seems to avoid this problem. When taken orally, bromelain tolerates gastric acid and is rapidly absorbed in its active form throughout the small intestine, forming a complex with anti-proteases 2-macroglobulin and 1-trypsin [5,41,45,46]. However, despite its glycosylated structure, bromelain can be modified in the digestive tract, particularly in the stomach, when ingested with certain foods [47]. It remains biologically active with a peak about one hour after ingestion and an absorption equal to 40% of the dose taken and with a plasma half-life of 6–9 h [45,48].

In terms of dosage, it has been shown that 160 mg/day of bromelain can have measurable beneficial effects, but it is hypothesized that more generous amounts (up to 750–1000 mg/day) are necessary to achieve optimal results. According to the literature, the effective dose is between 200 and 500 mg per administration. However, a maximum daily dose has never been established. It is usually recommended to divide the intake of the compound into several daily administrations [1].

In addition to dosage, it is also important to consider the proteolytic activity of the extract as measured in Gelatin Digestion Unit (GDU). All bromelain supplements are titrated in GDU/g: the higher the number, the more effective the enzyme. For effective supplementation, it is advisable to choose products with a GDU of at least 2000–2500 GDU [49].

Laboratory studies show that bromelain may inhibit some isoforms of cytochrome P450 and thus may cause a possible, although not directly demonstrated, increase in the bioavailability of some drugs *in vivo*. Bromelain increases bioavailability and reduces side effects associated with several antibiotics. It can increase the blood concentration of antibiotics such as tetracyclines and amoxicillin, explaining the effects of bromelain in the adjunctive treatment of some bacterial infections in patients undergoing antibiotic therapy [32,50]. In addition, other studies have suggested that bromelain may increase the absorption of chemotherapy drugs (such as 5-fluorouracil and vincristine), blood pressure medications (especially ACE inhibitors), drugs that induce drowsiness (such as lorazepam and diazepam), opioids (including codeine), and barbiturates (such as phenobarbital) [51]. There is no evidence on the effects of bromelain at higher concentrations, either in combination with other drugs or over long periods of time [47].

### 4.1. Toxicity and Side Effects

Animal studies have demonstrated that bromelain has very low toxicity, with a lethal dose (LD) of more than 10 g/kg when administered orally [22]. Toxicity studies in dogs receiving increasing doses of bromelain up to 750 mg/kg daily documented no toxic effects after six months. No cytotoxic or carcinogenic effects were observed in rats and dogs treated with the extract [41]. Although bromelain is well tolerated and considered a safe nutraceutical, clinical trials have reported some side effects, including temporary diarrhea, nausea, vomiting, allergic reactions, and risk of bleeding, especially in people treated with anticoagulants such as warfarin and clopidogrel [1]. Accordingly, bromelain is not recommended for patients taking anticoagulants or antiplatelet agents to avoid potential adverse events due to a possible additive effect. However, the effects of this substance on platelet function and blood coagulation are not fully understood, and there are studies in mouse models that have found a paradoxical tendency to hypercoagulability. These conflicting results may be partly explained by the limited sample size and the dosage of bromelain supplementation. In fact, Errasti et al. [52] highlighted the dual effect of bromelain on blood coagulation: at low doses, this supplement showed a procoagulant effect, while at high doses, it had an anticoagulant effect.

In clinical trials investigating the effects of bromelain on osteoarthritis, no serious adverse effects were found, although there were several cases of gastrointestinal symptoms, dry mouth, headache, fatigue, rash, and unspecified allergic reactions. In these trials, bromelain was given at doses ranging from 540 to 1890 mg daily [51]. Information regarding safety in pregnancy and lactation is lacking.

### 4.2. Methods of Extraction, Purification, and Stabilizing

There are currently no guidelines for the purification of bromelain, which can be performed through a variety of methods. The most appropriate technique to use depends on the intended application of the purified enzyme. For commercial use, bromelain is prepared by centrifugation, ultrafiltration, and lyophilization [2,53]. Its composition varies depending on the purification method and the source. It is extracted from the pineapple when the fruit is ripe, which is when the highest concentration of proteases is reached. Stem bromelain has a high protease content compared to fruit-derived bromelain [3,41,45]. Bromelain is susceptible to denaturation after lyophilization and loses approximately 50% of its initial activity. Lyophilization creates a number of stresses that tend to destabilize or unfold/denature unprotected proteins. This is due to disruptions in the hydration layer that occur when the water around the protein is extracted into a matrix of ice crystals. Therefore, stabilizers (such as cryoprotectants) are often required in protein formulations to maintain protein stability during the freezing and drying process, thereby minimizing denaturation [2].

Although conventional methods are commonly used, they often have limitations, particularly in terms of yield, purification, and processing costs. Modern techniques allow the use of traditional methods in combination with new approaches such as gel filtration, aqueous two-phase extraction, reverse micellar extraction, ion exchange, and affinity chromatography [6].

Conventional methods can still be used in pre-purification steps to concentrate and increase bromelain recovery. Once an adequate concentrate is obtained, subsequent purification steps to remove remaining impurities and increase final purity can be performed using modern techniques [2,3].

Therefore, a combination of low-resolution (filtration and liquid–liquid extraction) and high-resolution (chromatography) techniques seems to be the best approach to contain costs, provide high yields and low-impurity extracts, and preserve enzymatic activity [6]. Chromatographic techniques are the most promising because bromelain is primarily used in medical applications that require a high degree of purity [54].

Bromelain is sensitive to conditions such as high acidity, elevated temperature, gastric proteases in the stomach juice, chemicals, and organic solvents [4]. The instability of this enzyme under stress conditions limits its health benefits and pharmacological applications [55], hence the need for devising new techniques and improving already existing methods for its stabilization. Bromelain has been stabilized by immobilization on various nanoparticles [51], on carboxymethylchitosan of linoleic acid, on microspheres of chitosan, with N-isopropylacrylamide, and by binding to a Sepharose support matrix pre-coupled with concanavalin A. In addition, encapsulation of bromelain in katira gum nanoparticles was found to increase its stability [55]. To find edible matrices and technologies more suitable for bromelain stabilization, Nwagu et al. [56] successfully used spores of probiotic *Bacillus* spp. for immobilization of bromelain. They observed that immobilized bromelain also had significantly higher thermal and storage stability than free bromelain.

## 5. Use of Bromelain in Children and Adolescents

The main clinical indications for the use of bromelain in children and adolescents are shown in Table 1.

### 5.1. Upper Respiratory Tract Infections

Bromelain has shown beneficial anti-inflammatory and mucolytic properties in *in vitro* studies. It has been documented to have a significant effect on bronchial secretions, with the advantages of being a food ingredient and therefore safe for use in children [57,58,74,75,76,77].

Preparations based on *Ananas comosus* extract and honey containing proteolytic enzymes such as bromelain, trypsin, and chymotrypsin have shown promising results [1,16]. In the double-blind, randomized, parallel-group study by Peixoto et al. [57], children aged 2 to 15 years with irritative cough, were treated with a combination of *Ananas comosus* extract and honey (Bromelin^®^) compared to a placebo group receiving honey alone. They found a decrease in cough episodes in the two groups after 30 min of drug or honey administration. There were no additional effects of the combined preparation in the treatment of irritative cough. No adverse events were found.

It should be noted that a potential effect of bromelain, if any, would be more properly evaluated by considering another substance as a placebo, since honey may be a pharmacologically active agent in cough. In the respiratory setting, bromelain has been shown to have good serum and rhinosinusal tissue distribution when administered as a tablet to subjects affected by chronic rhinosinusitis, with symptom relief. [78,79]. It represents an adjunctive treatment for rhinosinusitis with more suggestive evidence of efficacy in acute rather than chronic forms, also in the pediatric population [59,60,61,62].

The activity of bromelain in the treatment of acute sinusitis was assessed in a multicentric, epidemiological cohort study including 116 children. The mean duration of symptoms was 6.66 days in subjects treated with bromelain extract (Bromelain-POS^®^) alone (monotherapy verum group), 9.06 days in children who received a combination of Bromelain-POS and standard therapy (combination therapy group), and 7.95 days in children treated with standard therapy alone (control group). Patients in the monotherapy verum group had a significantly faster symptom recovery (*p* = 0.005) as compared to the other treatment groups [58].

More recently, a case-control study evaluated the beneficial effects of an oral supplement (Flogostop Forte^®^) containing bromelain, Ribes nigrum, Boswellia serrata, and vitamin D in 60 subjects (including children >12 years) with chronic sinusitis. They were divided into a control group using nasal steroids only, with the treatment 1 and 2 groups using nasal steroids plus one dose of the oral supplement for 30 days and two doses of the oral supplement daily for 15 days, respectively. Local nasal inflammation (mucosal hyperemia and rhinorrhea) was significantly improved in patients treated with the supplement compared to controls [63].

In addition to its use as a complementary therapy for nasal and sinus swelling, bromelain has also been used for many non-respiratory conditions due to its anti-inflammatory effects. A randomized controlled trial investigated the effects of canned pineapple intake on the physical health, immune modulation, and nutritional status of ninety-eight schoolchildren (mean age: 8.44 ± 0.20). The first group consisted of subjects who did not receive pineapple, the second group included those who received 140 g, and the third group included those who received 280 g of pineapple for nine weeks. The results of this study documented a reduction in the incidence of viral and bacterial infections and an increase in granulocyte production (0.77–26.61%) in both groups that consumed pineapple [64]. 

A recent prospective case-control study involving a total of 120 children evaluated the efficacy of using a nutraceutical in oral solution (Flogostop^®^ Duo) containing 150 g of bromelain and Boswellia serrata in the treatment of upper respiratory tract inflammation and middle ear-related problems. The subjects were divided into three groups: both groups 1 and 2 were treated for 20 and 35 days, respectively, in combination with the standard treatment, while the control group received only standard treatment (nasal aerosol with fluticasone and mucolytic and nasal rinsing with hypertonic solution). This study showed a significant improvement in auditory, otoscopic, and fibroscopic findings (reduction in inflammation and viral spread in the upper airways) in group 2, 35 days after treatment initiation compared to the control group. This resulted in a better resolution of upper respiratory tract inflammation with a positive impact on patient well-being (Powell’s questionnaire) [65]. 

Bromelain may be helpful in the prevention of SARS-CoV-2 respiratory infection and may represent a complementary treatment for COVID-19 patients. This virus is able to infect the host cell by receptor-mediated endocytosis through the membrane-bound aminopeptidase angiotensin-converting enzyme II receptor (ACE2). SARS-CoV-2 has disulfide bridges for stabilization, which are crucial for binding to the ACE2 receptor in host cells [4]. This makes them a target for bromelain, which is able to break disulfide bonds and degrade proteins. Moreover, when bromelain was combined with N-Acetyl Cysteine (NAC), an increase in disulfide bond reduction was observed, resulting in inhibition of SARS-CoV-2 infectivity [80].

A recent study assessed the use of bromelain and NAC combination (BromAc^®^) in tracheal aspirate specimens from critically ill COVID-19 patients under mechanical ventilation. The authors found that this drug exerted a potent mucolytic effect on COVID-19 sputum *ex vivo* in a dose-dependent manner. In addition, they observed anti-inflammatory activity with a decrease in the effect of cytokine storm, regulatory cytokines (IL-5, IL-10, IL-13, IL-1Ra), and chemokines (MIP-1alpha, CXCL8, MIP-1b, MCP-1, and IP-10) compared with NAC alone and the control group [81]. Selected studies focusing on the use of bromelain alone or in combination for the treatment of respiratory infections in children are shown in Table 2.

### 5.2. Systemic Infections (Sepsis)

Proteolytic enzyme therapy based on bromelain may modulate the host immune system and promote early recovery from sepsis. Bromelain has been found to inhibit the effects of critical cytokines and molecules during the induction of inflammation by lipopolysaccharide (LPS) stimulation. LPS is an endotoxin from Gram-negative bacteria inducing inflammation and an excessive immune response, such as that seen in sepsis [82,83]. 

A phase III, double-blind, randomized, controlled trial in India evaluated the efficacy and safety of an oral enzyme preparation (Phlogenzym) containing 90 mg of bromelain, 48 mg of trypsin, and 100 mg of rutin as an adjuvant therapy for sepsis. Sixty children (ages 1 month to 12 years) received this formulation for 14–21 days along with standard treatment based on appropriate antibiotics and supportive care. The study results showed that the need for hemodynamic support, fever, and other clinical parameters normalized significantly earlier with faster recovery in the Phlogenzym group compared with the placebo group [34]. 

### 5.3. Dental Disorders

Bromelain is very effective in the relief of swelling and post-surgical edema in oral procedures and is also proposed for the therapy of caries and periodontal disease to prevent side effects of pharmacological treatment [8].

In children with dental fear, caries removal using conventional instruments such as the Airotor and high-speed handpiece is often a challenge. Chemo-mechanical caries removal is a minimally invasive technique that uses a chemical agent to remove infected dentin. Chemo-mechanical caries removal with organic gels is advantageous because it is less invasive, has fewer side effects, and is cost-effective. A recent study evaluated bromelain as a chemo-mechanical caries removal agent and compared it with papain in terms of efficacy. The mean of residual carious dentin thickness was much lower in the bromelain group compared to the papain group (36.74 μm vs. 73.84 μm), while the mean time required to remove carious dentin was found to be similar in the two groups (335.30 s vs. 352.33 s). The authors concluded that bromelain was more effective than papain in the amount of caries removal [66]. 

Wisdom tooth extraction appears to be a well-established indication for the bromelain treatment, and several studies support its efficacy in this field in both adolescents and adults [67,68,69,70]. In a study by Bormann et al. [69], patients aged 15 to 40 years undergoing surgery with fully retained wisdom teeth and an indication for osteotomy were divided into two groups and treated with bromelain and placebo, respectively. The analysis, which evaluated postoperative pain, swelling, and difficulty in swallowing, documented the efficacy of bromelain. In addition, this study showed that the efficacy of the treatment was not dose-dependent (3000 FIP and 4500 FIP vs. 1000 FIP, respectively).

The anti-inflammatory effects of bromelain after surgical extraction of a lower third molar have been evaluated in some studies in terms of reduction in postoperative pain and facial swelling and improvement in mouth opening. A study by Majid and Al-Mashhadani [68] compared the effects of oral bromelain (4 × 250 mg) versus oral diclofenac sodium (4 × 25 mg) on pain, swelling, trismus, and quality of life in 45 patients aged 18–35 years. The authors found that the effects of bromelain were comparable to those of diclofenac sodium for all parameters studied. More recently, Wala et al. [70] studied 230 patients undergoing lower third molar surgery and compared the anti-inflammatory properties of the combination of bromelain, trypsin and rutoside to the combination of ibuprofen, trypsin, and chymotrypsin. The group of patients treated with the drug combination including bromelain had a significant reduction in pain score compared with the group of patients receiving the other drug combination. The authors concluded that bromelain is an even superior analgesic compared to ibuprofen, so it could be considered an effective alternative option to NSAIDs for reducing postoperative complications.

Finally, the use of bromelain has also been proposed as a potentially effective tooth whitening agent due to its enzymatic action [84].

### 5.4. Burns and Associated Complications

Bromelain is a viable alternative to surgical escharotomy for deep burns [47]. In fact, prompt removal of necrotic tissue leads to better spontaneous healing or better graft application, reducing the risk of infection and improving patient survival [71]. Infection is the leading cause of death in burn patients, especially in children, due to the compromised skin barrier. In addition, the risk of infection persists until necrotic tissue remains [71]. Therefore, early eschar debridement within 24 h of the burn significantly reduces the risk of bacterial colonization and infection, especially in pediatric patients. However, this procedure can be associated with several complications, such as profuse bleeding with hemodynamic disturbances [72] or excision of the fascia, leading to significant skin deformity. Therefore, non-surgical eschar removal (abrasive techniques, hydrosurgery, or enzymatic debridement) is useful to minimize the risks associated with this procedure [71]. Claes et al. [72] found that a bromelain-based debridement agent can selectively remove the necrotic eschar while preserving viable dermal tissue and appendages in the wound bed in deep partial- or full-thickness margins; this results in spontaneous re-epithelialization and improved aesthetic and functional outcomes, especially when compared to tangential excision. This topical agent, called anacaulase-bcdb (NexoBrid^®^), contains several proteolytic enzymes enriched with bromelain. It was approved by the European Medicines Agency (EMA) in 2021 for eschar debridement [71] and by the US Food and Drug Administration (FDA) in 2022 [85].

Several studies have demonstrated the efficacy of NexoBrid in selectively removing burn eschar in a single 4-hour application, thus reducing the necessity for surgical excision of necrotic tissue [73] by at least 3.5 times [71] and preserving dermal tissue [72]. In addition, NexoBrid provided faster wound healing with better long-term outcomes [73] and improved quality of life [72]. One of the most important advantages is the selective action of NexoBrid with intact underlying tissue: this is useful for thin skin in children [73] and for deep and full-thickness burns where the depth of the lesion can be difficult to determine [71], reducing the risk of bleeding and hemodynamic instability [72]. In partial-thickness burns, it has resulted in spontaneous re-epithelialization, reducing graft risk, improving functional and aesthetic outcomes [72], and reducing hospital costs [71].

The biggest barrier to the use of bromelain is the lack of knowledge about pain management when applying the procedure. In fact, like surgery, NexoBrid treatments can induce hyperalgesia by releasing excitatory amino acids [72]. Claes et al. [72] found that locoregional anesthesia can provide pain relief while reducing the risks associated with general anesthesia, especially in the pediatric population or in patients with single limb involvement. For the lower limbs, epidural anesthesia was chosen instead of general anesthesia for two or more involved parts. In addition, with the use of bedside locoregional anesthesia, there was no need to transport the patient to the operating room for general anesthesia [72]. 

Some studies have confirmed the safety of NexoBrid in the treatment of adult and pediatric burns [71], while Shoham et al. [73] showed better results in children than in adults. Given the higher prevalence of burns in children (approximately 60%) and the important emotional and health significance of this condition, the use of enzymatic debridement for early eschar removal in the pediatric population is an important challenge to achieve a good long-term outcome [73]. 

## 6. Conclusions and Future Perspectives

Bromelain has attracted considerable industrial and medical interest. It has been used to treat various health problems but is also a popular dietary supplement. Its mechanism of action does not appear to be limited to proteolytic activity as a digestive enzyme but extends to a range of effects at different levels.

One of the main barriers to the widespread clinical use of bromelain is the variability in the composition of extracts, which leads to heterogeneous results and difficulty in formulating appropriate prescriptions. In fact, extracts with different activities can be obtained depending on the geographical location, the type of cultivation, and the extraction methods used. In addition, new techniques to improve the purification and extraction of bromelain are promising but still expensive and challenging. Recombinant DNA technology appears to be particularly promising, as recombinant bromelain has shown higher thermal stability and increased antimicrobial potency compared to the traditional commercial product [6].

In the pediatric population, the addition of bromelain to standard therapy may be beneficial, at least for the treatment of common upper respiratory tract infections, specific dental conditions, and burns. The ability of bromelain to enhance the efficacy of antibiotics while reducing their side effects should be targeted for use in children, where several infections could be treated more quickly by using this compound simultaneously with antibiotic therapy [51]. It has also been shown to be an effective alternative option to NSAIDs for reducing postoperative complications such as pain, swelling, inflammation, and risk of bleeding. In animal models, bromelain has also shown relevant activity in allergic respiratory diseases, including asthma [17,86], and in inflammatory bowel disease [19,87]. 

Further studies should better elucidate the action mechanism and functional properties of bromelain. At the same time, extraction and purification methods should be optimized. Although this compound has been used in pediatrics as a complementary therapy for several conditions, its interactions with various drugs, supplements, and foods need to be further investigated, and recommendations for its clinical use are lacking. Finally, new RCTs in pediatric settings are needed to better understand the effects of bromelain in different therapeutic areas.

## Figures and Tables

**Table 1:** Main clinical indications for the use of bromelain in children and adolescents.

Clinical Indication
Upper Respiratory Tract infections Adjuvant treatment of cough [57] Acute and chronic rhinosinusitis [58,59,60,61,62,63]
Recurrent upper respiratory tract infections [64] Nasal swelling with adenoid hypertrophy [65] Middle ear-related problems [65] Adjuvant therapy for sepsis [34] Oral surgery and dentistry Chemomechanical caries removal [66] Post-operative edema and pain [67,68,69,70]
Burns Eschar debridement [71,72,73]

**Table 2:** Selected studies focused on the use of bromelain alone or in combination for the treatment of respiratory infections in children and adolescents.

Author, Year [ref.]	Study Design	Study Population	Type of Disease Treated	Results
Peixoto et al., 2016 [57]	pragmatic, double-blind, randomized, parallel-group study	60 children aged 2–15 years	irritative cough (for at least 24 h)	The rate of cough improvement was similar in patients treated with pineapple comosus extract + honey (Bromelin^®^) compared to those receiving honey alone (placebo).
Braun et al., 2005 [58]	multicentric, epidemiological cohort study	116 children aged < 11 years	acute sinusitis	The monotherapy group (verum) had a shorter mean symptom duration (6.66 days) and significantly faster symptom resolution (*p* = 0.005) than the other groups.
Buttner et al., 2013 [78]	prospective, open-label observational pilot study	12 patients aged > 25 years	CRS (with or without nasal polyps) previously treated with sinus surgery	Adjunctive treatment with bromelain had positive effects on symptom reduction and quality of life.
De Luca et al., 2023 [63]	prospective case-control study	60 patients (children > 12 years adolescents and adults)	chronic sinusitis without nasal polyps (according to clinical EPOS classification)	Bromelain, Ribes nigrum, Boswellia serrata (Casperome^®^), and vitamin D added to standard topical treatment of chronic sinusitis may reduce mucosal inflammation and improve symptoms.
Cervo et al., 2014 [64]	randomized controlled trial	98 schoolchildren	Evaluation of effects of canned pineapple on the physical health, immune modulation, and nutritional status	In both groups that consumed pineapple, the incidence of infection was reduced, with increased granulocyte production.
Della Volpe et al., 2022 [65]	prospective case-control study	120 children aged > 12 months	OME and conductive hearing loss	Auditory, otoscopic, and fibroscopic findings improved in the group treated for 30 days compared to the control group, with a positive impact on patient well-being.
Coelho Dos Reis et al., 2022 [81]	blinded crossover study	20 COVID-19 patients aged ≥ 18 years	Critical COVID-19 (requiring mechanical ventilation)	Strong mucolytic effect of bromelain on COVID-19 sputum and anti-inflammatory activity.

CRS: chronic rhinosinusitis. OME: otitis media with effusion. EPOS: European Position Paper on Rhinosinusitis and Nasal Polyps.

## Data Availability

Not applicable.
